# Altered Germination and Subcellular Localization Patterns for PUB44/SAUL1 in Response to Stress and Phytohormone Treatments

**DOI:** 10.1371/journal.pone.0021321

**Published:** 2011-06-27

**Authors:** Jennifer N. Salt, Keiko Yoshioka, Wolfgang Moeder, Daphne R. Goring

**Affiliations:** 1 Department of Cell and Systems Biology, University of Toronto, Toronto, Ontario, Canada; 2 Centre for the Analysis of Genome Evolution and Function, University of Toronto, Toronto, Ontario, Canada; University of Melbourne, Australia

## Abstract

**Background:**

In plants, the ubiquitin-proteasome system is emerging as a significant regulatory system throughout the plant lifecycle. The ubiquitination of a target protein requires the sequential actions of the E1, E2 and E3 enzymes, with the latter E3 enzyme conferring target selection in this process. There are a large number of predicted E3 enzymes in plant genomes, and very little is known about the functions of many of these predicted genes. Here we report here an analysis of two closely-related members of the *Arabidopsis* Plant U-box (PUB) family of E3 ubiquitin ligases, PUB43 and PUB44.

**Principal Findings:**

Homozygous *pub44/pub44* mutant seedlings were found displayed a seedling lethal phenotype and this corresponded with widespread cell death lesions throughout the cotyledons and roots. Interestingly, heterozygous *PUB44/pub44* seedlings were wild-type in appearance yet displayed intermediate levels of cell death lesions in comparison to *pub44/pub44* seedlings. In contrast, homozygous *pub43/pub43* mutants were viable and did not show any signs of cell death despite the *PUB43* gene being more highly expressed than *PUB44*. The *PUB44* mutants are not classical lesion mimic mutants as they did not have increased resistance to plant pathogens. We also observed increased germination rates in mutant seeds for both *PUB44* and *PUB43* under inhibitory concentrations of abscisic acid. Finally, the subcellular localization of PUB44 was investigated with transient expression assays in BY-2 cells. Under varying conditions, PUB44 was observed to be localized to the cytoplasm, plasma membrane, or nucleus.

**Conclusions:**

Based on mutant plant analyses, the *Arabidopsis* PUB43 and PUB44 genes are proposed to function during seed germination and early seedling growth. Given PUB44's ability to shuttle from the nucleus to the plasma membrane, PUB44 may be active in different subcellular compartments as part of these biological functions.

## Introduction

The ubiquitin-proteasome system is a major regulatory pathway in all eukaryotic organisms. Ubiquitination of a target is carried out by the sequential action of three enzymes: the E1 ubiquitin-activating enzyme, the E2 ubiquitin-conjugating enzyme, and the E3 ubiquitin ligase. Of these three enzymes, the E3 ubiquitin ligase is responsible for target selection, and in keeping with this role, there are a large number and variety of E3 ligases [1]. Once a target protein is selected by E3 enzyme and ubiquitinated, the main fate is degradation by the 26S proteasome resulting in the down-regulation of the target protein. Ubiquitination of larger complexes and organelles can also lead to another degradation pathway such as autophagy [2]. Finally, protein ubiquitination has been found to have important regulatory roles beyond protein degradation such as in transcription, DNA repair, and protein trafficking [3]. In plants, the ubiquitin-proteasome system has a variety of functions through the plant lifecycle [4]. Interestingly, analyses of gene families for components of this system have shown that there have been some significant gene expansions compared to other organisms, in particular for E3 ligases, suggesting a strong reliance on this regulatory system in plants. For example, there are >1400 predicted E3 ligase genes in the *Arabidopsis* genome compared to the >600 predicted E3 ligases estimated for the human genome [3], [4].

The Plant U-box (PUB) family represents one group of E3 enzymes with 64 predicted members in *Arabidopsis* [5], [6], [7]. Based on the structure of the *Arabidopsis* PUB14 U-box, this domain has been predicted to form an E2-binding domain and is proposed to bring the E2 in proximity to the E3-bound target for ubiquitination [8]. In combination with the U-box, there are other predicted domains present such as Armadillo (ARM) repeats, Ser/Thr kinase, WD40 repeats, tetratricopeptide repeats or peptidyl-prolyl isomerase domain [7], [9]. A large number of the PUB proteins are predicted to contain ARM domains with varying numbers of ARM repeats [6], [10], [11]. For several PUB proteins, the ARM domains have been previously found to interact with kinase domains [12], [13], [14], [15]. Members of the PUB-ARM subfamily have been implicated in a number of different plant processes including pollen-pistil interactions [12], [16], [17], [18], [19], flowering time [20], hormone responses [14], [21], [22], stress responses [23], [24], [25], [26], and plant-microbe interactions_ENREF_25 [15], [26], [27], [28], [29], [30], [31]. In this study, we present an analysis of T-DNA insertion mutant lines for two closely related *Arabidopsis* PUB-ARM genes, PUB43 and PUB44. Previously, Raab et al. [22] observed that *pub44 (saul1)* mutants were undergoing premature leaf senescence under low light conditions. They also linked PUB44/SAUL1 to abscisic acid (ABA) biosynthesis through the *Arabidopsis* Aldehyde Oxidase (AAO3) protein, and proposed a model where PUB44/SAUL1 negatively regulated leaf senescence by negatively regulating ABA levels. Here, we present additional altered phenotypes for the PUB44 T-DNA insertion lines during germination and seedling growth, and investigate the subcellular localization of PUB44 under different treatment conditions.

## Results

### Characterization of PUB44 and PUB43 T-DNA insertion lines

**Figure 1:**
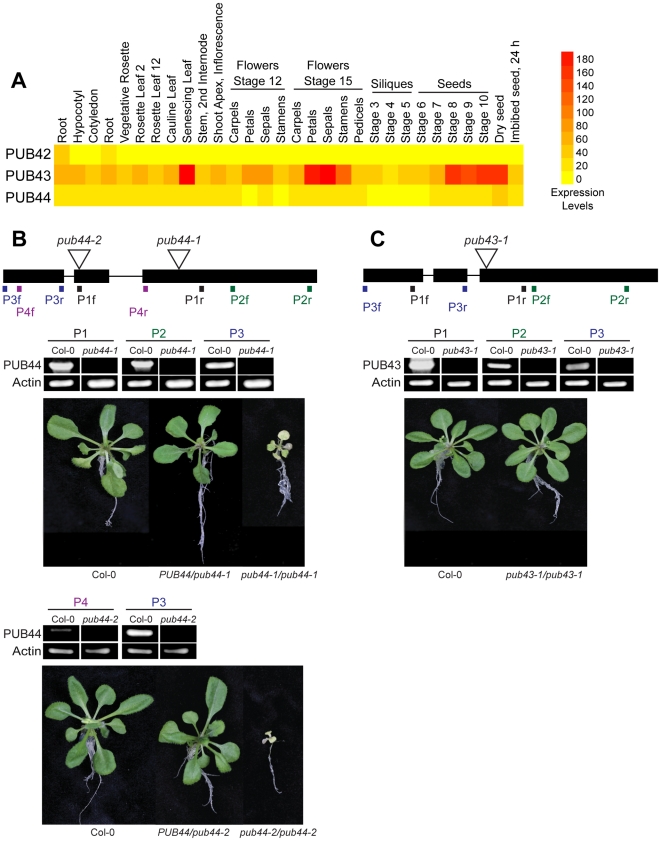
Microarray expression analyses and T-DNA insertion lines for the PUB44 and PUB43 genes. (**A**) Microarray expression analyses of the PUB42 (At1g68940), PUB43 (At1g76390), and PUB44 (At1g20780) genes in different tissues. E-northern analyses of the public microarray datasets were conducted through the BioArray Resource [35], [36], [52], [53]. (**B**) T-DNA insertion lines for the *PUB44* gene. A schematic representation of the PUB44 gene at the top shows the positions of two different T-DNA insertions: Sail_716_H08 (*pub44-1*) and SALK_076799 (*pub44-2*) (black boxes  =  exons; lines  =  introns). The *pub44-2* allele has also been reported as *saul1-2* [22]. The positions of primers used for the RT-PCR analyses are also shown. The centre panel shows the results of the RT-PCR analyses which indicate a loss of PUB44 expression in the T-DNA insertion lines. P1, P2, P3 and P4 represent the PUB44 primers used in each reaction. Col-0 cDNA was used as a positive control for PUB44 expression, and actin was used as a positive control for the RT-PCR reactions. The bottom panel shows three week old Col-0 *PUB44/PUB44*, *PUB44/pub44-1* and *pub44-1/pub44-1* plants. The *pub44-1/pub44-1* plants display a seedling lethal phenotype under standard growth conditions and do not survive to adulthood. (**C**) A single T-DNA insertion line for the *PUB43* gene. A schematic representation of the PUB43 gene shows the positions of the SALK_112870 T-DNA insertion (*pub43-1*) (black boxes  =  exons; lines  =  introns). The positions of primers used for the RT-PCR analyses are also shown. The centre panel shows the results of the RT-PCR analyses which indicate a loss of PUB43 expression in the T-DNA insertion line. P1, P2 and P3 represent the PUB43 primers used in each reaction. Col-0 cDNA was used as a positive control for PUB43 expression, and actin was used as a positive control for the RT-PCR reactions. The bottom panel shows three week old Col-0 *PUB43/PUB43* and *pub43-1/pub43-1* plants. No growth defects were observed in the *pub43-1/pub43-1* plants.

The *Arabidopsis* genome has 41 predicted genes belonging to the U-box/ARM repeat family of E3 ligases as previously described [6]. Within this group, there is a subclade of three predicted genes, *PUB42*, *43*, and *44*, which share a unique domain organization with an extended C-terminal ARM repeat region containing up to 12 ARM repeats [6]. A survey of the public microarray databases shows that PUB42 shows very low or no expression in most tissues surveyed (Figure 1A). In contrast, PUB43 and PUB44 mRNA are both detected in a range of tissues surveyed with PUB43 generally being expressed at higher levels (Figure 1A). A more limited survey of PUB44 expression by RNA blot analysis also showed this [6]. Thus, we focused on knocking out PUB43 and PUB44 functions in *Arabidopsis* to examine their overall effects on growth and morphology and identified T-DNA insertion lines for these genes. T-DNA insertions were identified in both the SAIL *pub44-1* and SALK *pub44-2*, *pub43-1* collections (Figure 1B and C).

In each case, heterozygous plants were first identified, allowed to self-fertilize, and the resulting progeny were screened to obtain homozygous mutant plants. RT-PCR was used to confirm that these insertions generated a complete loss of *PUB44* and *PUB43* transcripts (Figure 1B and C). While homozygous *pub43-1/pub43-1* plants could be readily identified, the *pub44/pub44* mutants were difficult to find and typically had a lethal phenotype (Figure 1B). This PUB44 (SAUL1) premature senescence phenotype was previously reported to be related to growth under low light levels 60 µmol m^−2^ s^−1^ and rescued by growth under very high light levels over 400 µmol m^−2^ s^−1^; [22]. We observed this phenotype when plants were grown on soil at light levels of 125 µmol m^−2^ s^−1^ under long day conditions. However, we did not observe this phenotype in the *pub43-1/pub43-1* plants, and a correct segregation of the expected 1∶2∶1 ratio was observed [n = 100 23∶51∶26; χ^2^ = 0.22, P<0.05].

### Cell death lesions are present in both heterozygous and homozygous pub44 mutants

**Figure 2:**
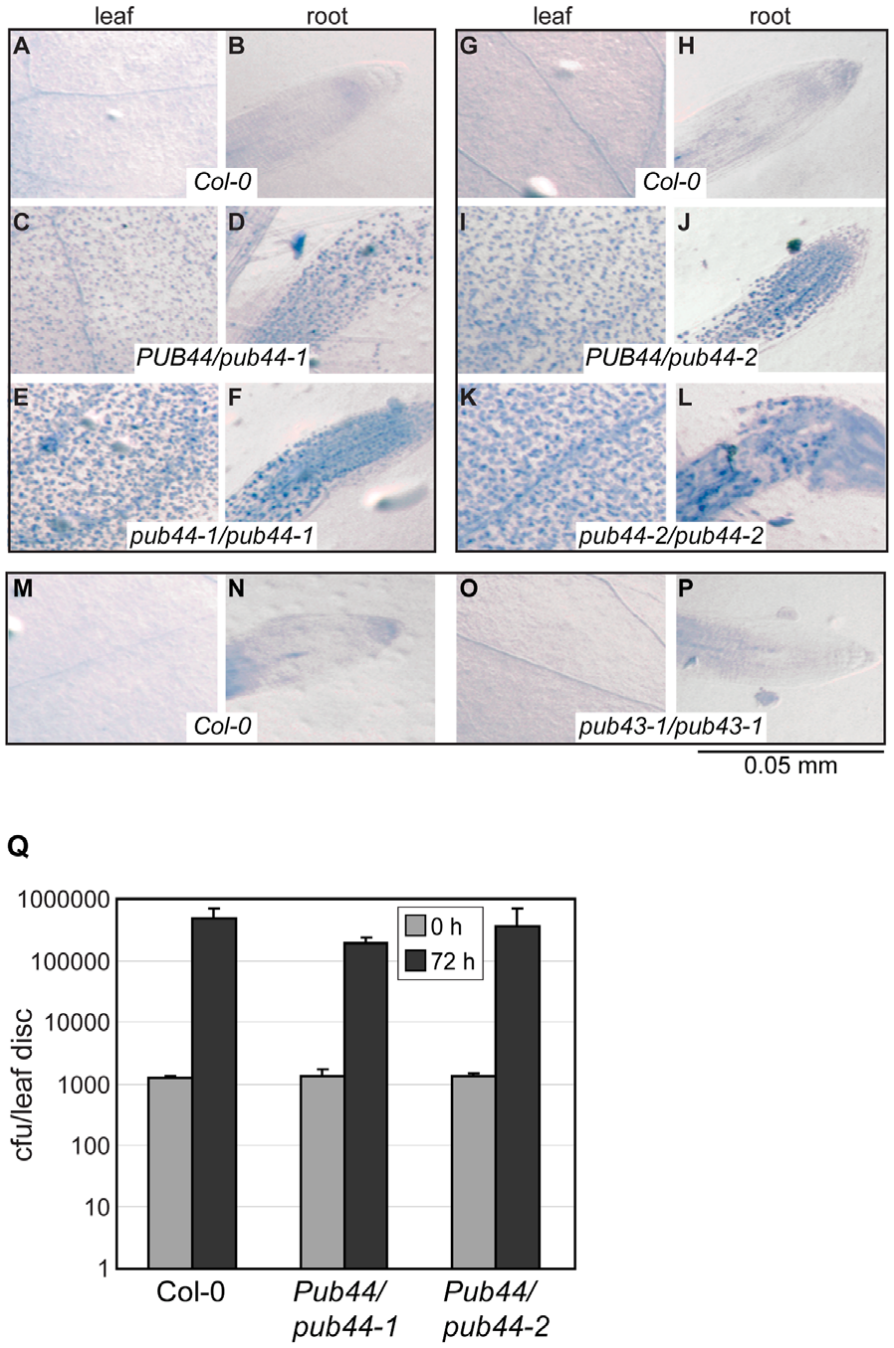
Cell death lesions in *PUB44/pub44* plants and responses to *Pseudomonas syringae pv*. *tomato* infections. (**A**–**P**) Trypan blue staining assay for cell death. 3 day old seedlings were stained with trypan blue to further investigate the seedling lethality phenotype associated with the *pub44/pub44* genotype. Images of stained cotyledons and roots are shown. Seeds from heterozygous *PUB44/pub44* plants were germinated to give seedlings with the three expected genotypes: Col-0 *PUB44/PUB44*, *PUB44/pub44*, and *pub44/pub44* determined by PCR genotyping. The *pub44/pub44* homozygous seedlings showed the greatest amount of trypan blue stained cell death lesions, and the *PUB44/pub44* heterozygous seedlings showed an intermediate amount of staining, despite being viable plants. In contrast, the *pub43-1/pub43-1* seedlings did not have any detectable cell death lesions. Scale bar = 0.05 mm. (**Q**) Responses of *PUB44/pub44* plants to pathogen infection. Four-week old Col-0 and *PUB44/pub44* plants were infected with *Pseudomonas syringae pv*. *tomato*, and bacterial counts were taken after 3 days. The *PUB44/pub44* plants showed a similar response to Col-0. Average bacterial counts were taken from plants and graphed with standard error.

To further investigate the cause of seedling lethality observed in the *pub44/pub44* mutants, trypan blue analysis was conducted to determine if increased levels of cell death could be observed, and responsible for the loss of the homozygous *pub44/pub44* mutants in older seedling populations. Seeds from heterozygous *PUB44/pub44* plants were germinated on plates with light levels of 75 µmol m^−2^ s^−1^ under long day conditions, and 3-day-old seedlings were collected and stained with trypan blue which only stains dead cells. The seedlings were a mixture of wild-type Col-0, heterozygous *PUB44/pub44*, and homozygous *pub44/pub44* plants, and while all the seedlings appeared wild-type in appearance, three distinct staining patterns were observed with the trypan blue (Figure 2A-P). PCR genotyping confirmed that the three distinct staining patterns corresponded to the three different genotypes [n =  100 27∶47∶26; χ^2^ =  0.38, P<0.05]. Distinct punctate staining patterns could be seen in the cotyledons and roots of *PUB44/pub44* heterozygotes (Figure 2C, D, I and J) and *pub44/pub44* homozygotes (Figure 2E, F, K and L) that were not present in wild-type Col-0 seedlings (Figure 2 A, B, G and H). As well, the intensity of staining was increased in the homozygotes (Figure 2E, F, K and L) compared to heterozygotes (Figure 2C, D, I and J). Thus, there was *pub44* gene dosage effect for the appearance of cell death lesions. While this eventually causes a lethality of the *pub44/pub44* homozygotes, the heterozygous *PUB44/pub44* plants did not show any morphological changes (Figure 1B). However, when homozygous *pub43/pub43* seedlings were tested, no cell death lesions were observed (Figure 2M–P).

It has been shown for other cell death mutants, that the presence of increased cell death lesions is correlated with an increased protection against pathogen infection [32]. As well, several other members of the PUB-ARM family have been implicated in plant pathogen responses [33]. For examples, a rice recessive mutant, *spl11*, was previously found to be a lesion mimic mutant that had strong non-race-specific resistance to both the rice blast fungus and the bacterial blight pathogen [27], [34]. Thus, we were interested in investigating if the *PUB44/pub44* plants displayed altered responses to pathogen infections. Progeny from heterozygous *PUB44/pub44-1* and *PUB44/pub44-2* parental lines, along with the control Col-0 plants, were infected with *Pseudomonas syringae pv*. *tomato* DC3000, a strain that is virulent on Col-0 plants. The infections were conducted on 4 week old plants where the homozygous *pub44-1/pub44-1* and *pub44-2/pub44-2* seedlings would have already died off and only the wild-type and heterozygous *PUB44/pub44* genotypes are present in a 1∶2 ratio. Nevertheless, all plants that were tested permitted a similar degree of bacterial growth at 72 hours following infection (Figure 2Q). Additionally, 7-day-old seedlings from the heterozygous *PUB44/pub44-1* and *PUB44/pub44-2* parental lines were infected with the virulent oomycete pathogen *Hyaloperonospora arabidopsidis* isolate, Noco2. The presence of sporangiophores on the cotyledons was scored at 7 days post inoculation Table 1. At this stage, the *pub44/pub44* mutant seedlings were dying off and could not be assessed for disease resistance as *Hyaloperonospora arabidopsidis* is a biotroph. The viable seedlings, which were a mixture of wild-type and heterozygous *PUB44/pub44* genotypes in a 1∶2 ratio were scored, and again, the percentage of susceptible plants was comparable to that seen for the Col-0 control plants (Table 1). As well, no differences were observed between the *PUB44/pub44* and Col-0 seedlings in the number of sporangiophores present per cotyledon. Taken together, these data suggest that the heterozygous *PUB44/pub44* plants do not have enhanced disease resistance under our test conditions even though a mild lesion mimic-like phenotype was observed with trypan blue staining.

**Table 1:** Infection rates of *Hyaloperonospora arabidopsidis* isolate Noco2 at 7 days after infection on Col-0 seedlings or seedlings from *PUB44/pub44* parental plants.

Parental Genotype	Total	Viable	Lethal a	Infected b	% Infection b
Col-0	89	89	0	79	89%
*PUB44/pub44-1*	47	36	11	34	94%
*PUB44/pub44-2*	43	36	7	32	88%

a Seedling death due to *pub44/pub44* phenotype.

b Viable seedlings only as this pathogen is a biotroph.

### Germination responses under stress conditions

**Figure 3:**
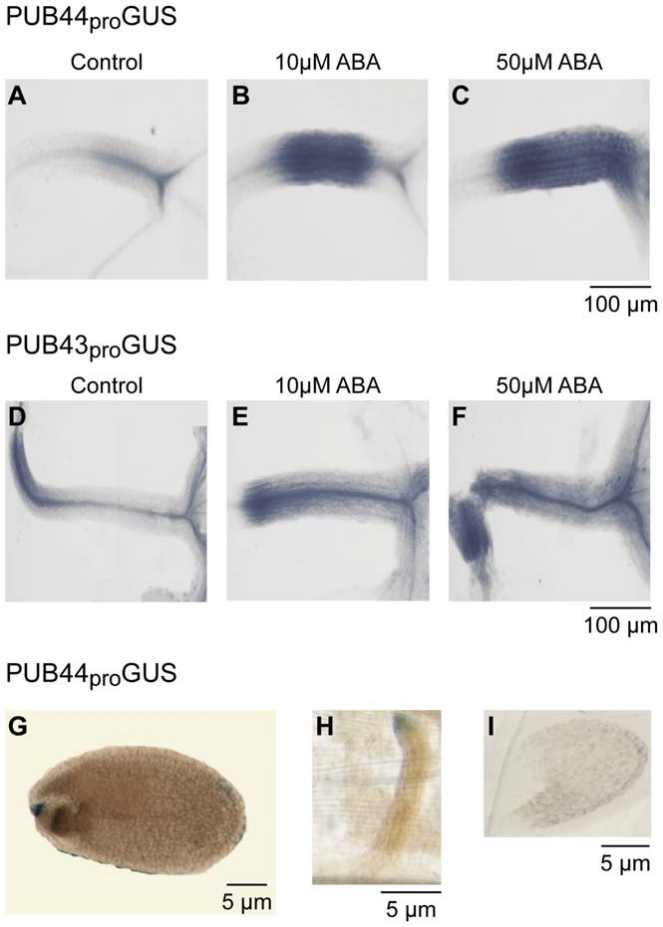
GUS activity staining of PUB44_pro_GUS and PUB43_pro_GUS transgenic plants. (**A**–**F**) GUS activity following treatment of 7 day old seedlings with ABA. Seedlings were treated with 10 µM ABA or 50 µM ABA for 2 hours. Images of the hypocotyls are shown. A minimum of 10 seedlings and 3 replicate experiments were analyzed, and representative images are shown. (**G**–**H**) GUS activity in seeds from PUB44_pro_GUS transgenic plants. G seed, H funiculus, and I embryo. Scale bars represent distances as indicated.

**Figure 4:**
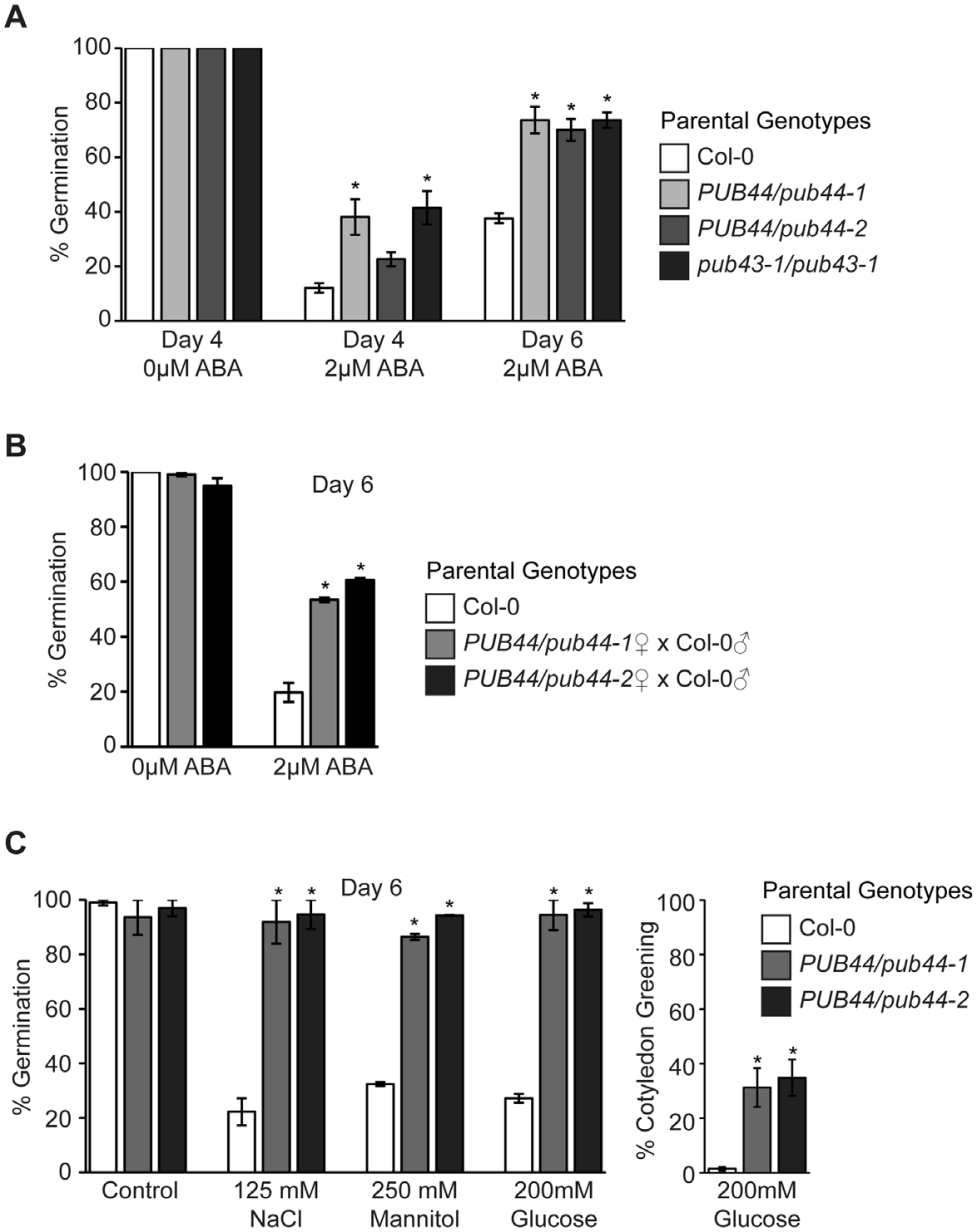
Responses of the pub44 and pub43 T–DNA lines to various stresses treatments during germination. (**A**) Seed germination rates at Days 4 and 6 for seeds planted on 0 or 2 µM ABA. Germination was scored when the radicle was fully emerged. Seeds from heterozygous *PUB44/pub44* and homozygous *pub43/pub43* plants showed decreased sensitivity to ABA during germination in comparison to Col-0 seeds. (**B**) Seed germination rates and segregation of the *pub44* allele in seeds with decreased sensitivity to ABA. Seeds from a *PUB44/pub44* ♀ x Col-0♂ cross were subjected to germination assays in the presence of 2 µM ABA and germinated seedlings were genotyped for the presence of the *pub44* allele. A 1∶1 ratio of wild-type and *PUB44/pub44* seedlings were observed in the germinated seedlings displaying decreased sensitivity to ABA [n =  100 47∶53; χ^2^ =  0.36, P<0.05]. (**C**) Seed germination rates for seeds from *PUB44/pub44* plants at Days 6 plated on 125 mM NaCl, 250 mM mannitol, or 200 mM glucose. Cotyledon greening was also assessed for the seedlings plated on 200 mM glucose. Decreased sensitivity was observed for seeds from *PUB44/pub44* heterozygous plants under all conditions. Asterisks represent statistically significant differences (P>0.05) when compared to appropriate Col-0 controls. n>50 with 3 replicates per assay.

A survey of the public microarray expression datasets (Bio-Array Resource site) [35], [36] for conditions associated with increased PUB44 expression generally revealed very little change for PUB44 under different treatments, though a four-fold increase was observed with NaCl treatment. Interestingly, Hoth et al. [37] used massively parallel signature sequencing to identify genes that are highly up-regulated with abscisic acid (ABA) treatment, and PUB44 was one of the candidate genes from this study. We tested whether the PUB44 and PUB43 promoters could respond to ABA treatment by generating transgenic *Arabidopsis* Col-0 plants carrying either a PUB44_pro_GUS or PUB43_pro_GUS construct (Figure 3). Transgenic seedlings with either GUS construct showed increased GUS activity with ABA treatment (Figure 3A–F). Thus, we conducted germination assays to test for altered responses in the presence of ABA. Seeds harvested from heterozygous *PUB44/pub44-1* and *PUB44/pub44-2* plants as well as homozygous *pub43-1/pub43-1* seeds were germinated in the presence of 2 µM ABA, a concentration that is known to inhibit germination (Figure 4A). Seeds from the heterozygous *PUB44/pub44* plants were able to germinate at levels that were significantly higher than that seen for Col-0 seeds. The *pub43/pub43* seeds also showed a similar insensitivity to 2 µM ABA (Figure 4A).

With the use of seeds from *PUB44/pub44* parental plants in these germination assays, the question arose as to whether the homozygous *pub44/pub44* seeds were largely responsible for the increased germination rates observed in the presence of 2 µM ABA. To address this question, *PUB44/pub44* pistils were pollinated with Col-0 pollen, and the resulting seeds were subjected to germination assays in the presence of 2 µM ABA. These seeds would be predicted to be a mixture of wild-type and heterozygous *PUB44/pub44* genotypes in a 1∶1 ratio, and in the presence of 2 µM ABA, increased germination rates were again observed (Figure 4B). Seedlings that germinated under these conditions were then genotyped for the *pub44* allele to determine the ratio of wild-type and heterozygous *PUB44/pub44* seeds in this population. A 1∶1 ratio of wild-type and *PUB44/pub44* seedlings was observed, and there was no bias towards seedlings containing the *pub44* mutant allele [n =  100 47∶53; χ^2^ =  0.36, P<0.05]. Therefore, the decreased sensitivity to the inhibitory effects of ABA during germination appears to result from the heterozygous *PUB44/pub44* maternal tissue during seed development. PUB44 expression can be detected at low levels in the developing seed in the public microarray databases (Figure 1A). Interestingly, the PUB44_pro_GUS transgenic plants had detectable levels of GUS activity in the seed coat, particularly at the attachment point for the funiculus (Figure 3G). The tip of funiculus also stained blue (Figure 3H), but no GUS activity was detected in the embryo (Figure 3I).

With the association of ABA with stress responses, we also investigated if the seeds from heterozygous *PUB44/pub44* plants showed altered germination rates in the presence of other germination stresses such as NaCl, mannitol, and glucose. In the presence of 125mM NaCl, 250mM mannitol, or 200mM glucose, seeds harvested from either heterozygous *PUB44/pub44-1* or *PUB44/pub44-2* plants had excellent germination rates while Col-0 seeds showed strong germination inhibition under these conditions (Figure 4C). Additionally, following germination on high concentrations of glucose, seedlings normally accumulate high levels of anthocyanins in the presence of glucose and are unable to green. When this trait was examined, seedlings from *PUB44/pub44* parental plants had increased percentages of cotyledon greening and thus, were able to produce chlorophyll whereas control Col-0 seedlings showed very little greening produced anthocyanins (Figure 4C).

### PUB44 subcellular localization in tobacco BY-2 cells

**Figure 5:**
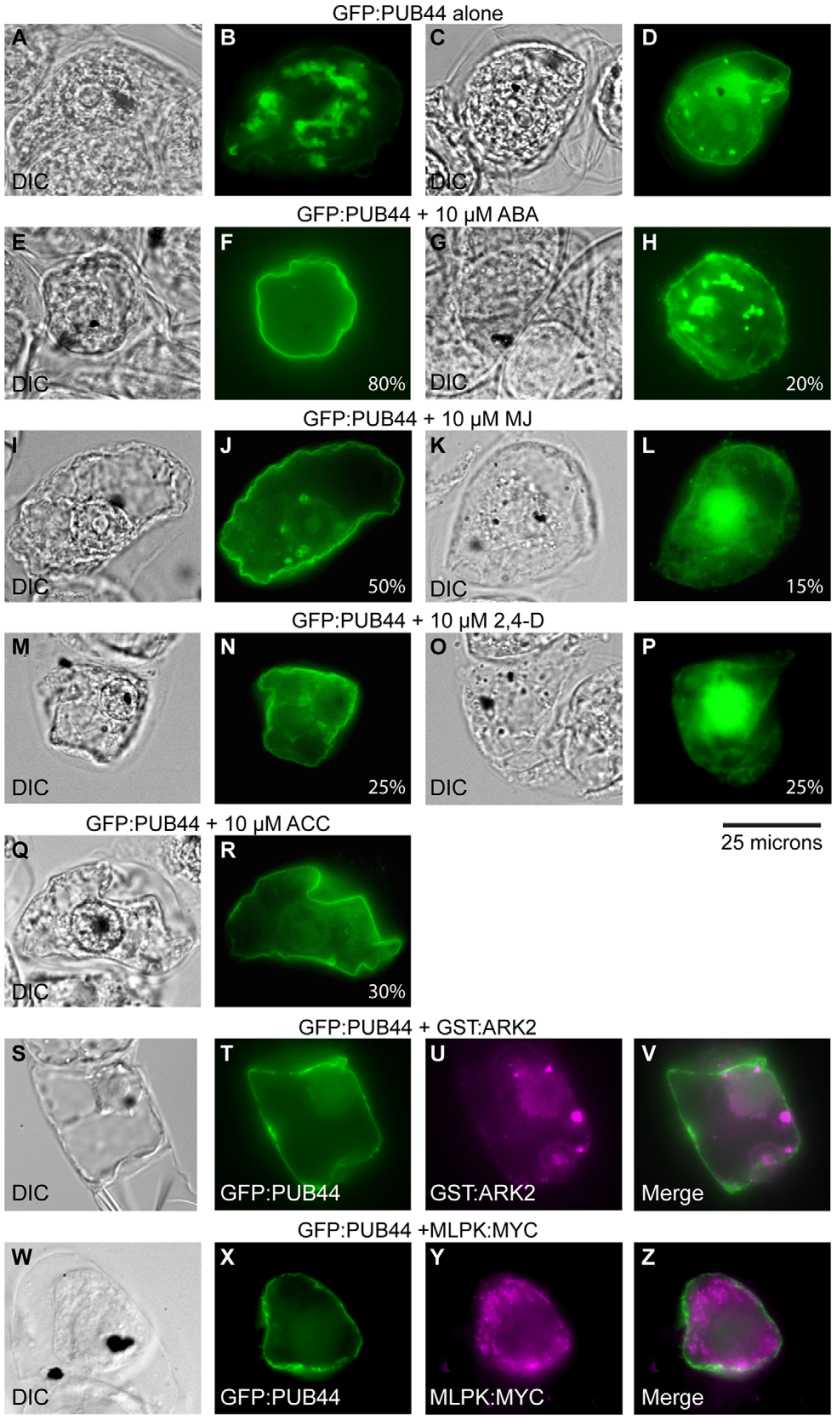
Transient expression and subcellular localization of GFP:PUB44 in tobacco BY-2 cells. Tobacco BY-2 cells were bombarded with the GFP:PUB44 construct, and the subcellular localization of pattern for PUB44 was determined at 18 hours after transformation. (**A**–**R**) The effects of phytohormone treatment on GFP:PUB44 localization after 2 hours of treatment. DIC and GFP fluorescent images are shown. GFP:PUB44 on its own is found in the perinuclear region of the cell with some also present at the plasma membrane B, D. Hormone treatments caused re-localization of GFP:PUB44 to the plasma membrane or the nucleus. The % transformants with a particular localization pattern are indicated on the bottom right of each panel. (**S**–**Z**) BY-2 cells co-expressing GFP:PUB44 with an active kinase. The GFP:PUB44 construct was co-bombarded with either the GST-ARK2 or MLPK:MYC constructs [14]_ENREF_27_ENREF_18. GFP fluorescence GFP:PUB44 is shown in green T,X while immunofluorescence patterns for GST-ARK2 or MLPK:MYC are shown in magenta U, Y. Areas of overlap in the merge images V and Z appear as white. The active kinases also redistribute GFP:PUB44 to the plasma membrane with some also in the nucleus. Scale bar represents 25 µM. Experiments were replicated a minimum of 3 times, with at least 20 cells per replicate showing the same distribution. Representative images are shown.

The PUB-ARM proteins have previously been found to sort to different subcellular compartments [14], [18], and with the cell death phenotype of the *pub44/pub44* mutants, we were interested to see where PUB44 could be found within the cell. PUB44 is predicted to be a cytosolic protein; however, it was identified in a plasma membrane proteomics dataset suggesting that it is plasma membrane localized [SUBA II database; [38], [39]. A PUB44:GFP fusion was transiently transformed by bombardment into tobacco BY-2 cells, and was found to be generally localized to perinuclear punctate structures (Figure 5A, B) with some also present at the plasma membrane (Figure 5C, D). Previously, we found that other PUB-ARM proteins, *Brassica* ARC1 and the *Arabidopsis* PUB9, were found to redistribute to different compartments in the presence of select active kinase domains or phytohormones [14], [18]. In the case of Brassica ARC1, a perinuclear localization pattern was observed in BY-2 cells when co-expressed with an active kinase domain from the *S* Receptor Kinase [18]. To see if PUB44 was similarly responsive to these treatments, several conditions were tested. Given the ABA germination responses observed, the effects of ABA on GFP:PUB44 subcellular localization was investigated by treating BY-2 cells transiently expressing GFP:PUB44 with 10 µM ABA for 2 hours (Figure 5E–H). A majority cells exposed to ABA and expressing GFP:PUB44 show a PUB44 primarily at the plasma membrane, with smaller percentage still showing some perinuclear-localised PUB44.

To determine if this effect was specific to ABA, four other phytohormones were also investigated: gibberellic acid (GA_3_), methyl jasmonate (MJ), 2,4-dichlorophenoxyacetic acid (2,4-D), and 1-aminocyclopropane-1-carboxylic acid (ACC). MJ treatment also resulted in the plasma membrane localization of PUB44, though at a lower frequency than that observed for ABA (Figure 5I, J). Interestingly, PUB44 was also found to localize to the nucleus in approximately 25% of the expressing cells (Figure 5O, P). Following treatment with 2,4-D auxin treatment, changes in PUB44 localizations patterns were only observed in about half the expressing cells, with GFP fluorescence at the plasma membrane or nucleus observed (Figure 5M–P). Treatment with ACC (ethylene) largely did not change the localization of PUB44, though plasma membrane localization was again observed in some cells (Figure 5Q, R). The addition of GA_3_ did not have any noticeable changes in the localization of PUB44.

Finally, the effects of two kinases, ARK2 and MLPK, previously tested on other PUB-ARM proteins [14] were also examined for PUB44. For ARK2, the cytosolic kinase domain is expressed as a GST:ARK2 fusion while full length MLPK has as C-terminal MYC tag. The co-expression of either the ARK2 kinase domain or MLPK with GFP:PUB44 resulted in the redistribution of PUB44 from the perinuclear region to the plasma membrane (Figure 5S–Z). There also appeared to be some GFP:PUB44 present in the nucleus as well (Figure 5S–Z). Overall, the more general changes in PUB44's localization patterns with either phytohormone treatment or co-expression of a kinase is unusual for what we have previous observed with PUB-ARM proteins, and perhaps indicates that PUB44 has a general cellular function in a number of different signalling responses.

## Discussion

In this study, we have characterized T-DNA insertion lines for two closely-related E3 ubiquitin ligase genes, *PUB44* and *PUB43*. Interestingly, the homozygous *pub44/pub44* seedlings displayed a seedling lethality phenotype while the *pub43/pub43* plants were wild-type in appearance. Raab et al. [22] also observed the same phenotype for the *pub44/pub44 (saul1)* mutants and identified that these seedlings were undergoing premature leaf senescence under low light conditions. Furthermore, they found that this phenotype in the *pub44/pub44* mutants was accompanied by decreased chlorophyll levels and photosynthetic activity, increased expression levels of a number of genes associated with the senescence and the autophagy pathway, and increased levels of ABA in leaves. Finally, they found that the *Arabidopsis* AAO3 protein, which catalyzes the last step in ABA biosynthesis, had increased activity in the *pub44/pub44* mutants and demonstrated an *in vitro* interaction between PUB44 and AAO3. From this, Raab et al. [22] proposed a model for PUB44/SAUL1 in negatively regulating leaf senescence by negatively regulating ABA levels through AAO3 activity.

In this study, we have presented evidence for PUB44 having additional functions in *Arabidopsis*. Using trypan blue staining, we observed cell death lesions in both the cotyledons and roots of three day old *pub44/pub44* seedlings which suggest that PUB44 may function as a negative regulator of cell death in biological functions beyond leaf senescence. As well, heterozygous *PUB44/pub44* seedlings were found to have cell death lesions, although to a lesser extent than that observed in the *pub44/pub44* seedlings. Yet, the *PUB44/pub44* plants were viable and visibly indistinguishable from wild-type Col-0 plants. Perhaps, the residual amount of PUB44 activity restricts cell death to a manageable level for the seedlings to recover. This phenotype is not a classical lesion mimic mutant [32] as these plants did not show any altered responses to pathogens as seen, for example, for the rice PUB mutant, *spl11* [27].

The presence of cell death in the heterozygous *PUB44/pub44* seedlings is suggestive of haploinsufficiency effects, where both functional copies of a gene are required to produce a wild-type phenotype. There are other examples of *Arabidopsis* ubiquitin pathway genes functioning in a dosage dependent manner such as the HECT-containing ubiquitin ligase, UPL3, affecting trichome branching [40] and the RING E3 ubiquitin ligase, Big Brother, regulating organ size [41]. The different levels of cell death lesions in the heterozygous *PUB44/pub44* and homozygous *pub44/pub44* plants suggest that the tight regulation of PUB44 levels is essential in maintaining proper regulation of cell death. The expression data for PUB44 suggests that its RNA levels are maintained at low levels over different conditions as seen by RNA blot analysis, microarrays, and PUB44_pro_GUS transgenic plants [6,22, this study]. In contrast, PUB43 is more highly expressed, and did not display any altered growth phenotype in this study suggesting that PUB43 may have a different pattern of regulation in leaf and root tissues.

Germination assays using seeds from heterozygote *PUB44/pub44* plants identified a role for PUB44 in inhibiting germination of seeds under conditions that are not favourable for growth. This included treatments with ABA, glucose, NaCl and mannitol where reduced levels of PUB44 allowed for seed germination under conditions that were inhibitory to wild-type Col-0 seeds. The *pub43/pub43* seeds also displayed increased germination rates with ABA treatment. For the seeds from *PUB44/pub44* plants, it appears that maternal tissues are responsible for some of the increased germination rates observed. Analysis of crosses between wild type and heterozygous plants demonstrates that maternally derived *PUB44/pub44* seeds had increased germination in the presence of ABA. This has been previously observed for other ABA mutants [42]. Support for maternal tissues affecting seed germination is also seen where PUB44_pro_GUS expression is detected in the funiculus and in the seed coat at the point of funicular attachment. It is unknown whether this is related to structural changes in these maternally-derived seed tissues, perhaps a cell death related developmental event resulting in seeds that do not respond normally to inhibitory germination cues.

The linkages with ABA are notable, given that Raab et al. [22] previously found increased levels of ABA in *pub44/pub44* mutant leaves. Our results reported here raise the possibility that PUB44 and PUB43 may function as part of the inhibitory effect of ABA on seed germination. However, further research is needed to better define the connection between PUB43/44 and ABA in the inhibition of seed germination. The increased germination in the presence of other germination stresses such as NaCl, mannitol, and glucose is also interesting as it may be linked to a more general role in abiotic stress responses. A number of PUB genes display increased expression during abiotic stress responses, and several mutants have been found to have altered growth under abiotic stress conditions [9], [23], [24], [25], [26]. For example, overexpression of the pepper PUB1 cDNA in *Arabidopsis* resulted in increased sensitivity to drought and salt [23]. The related *Arabidopsis PUB22* and *PUB23* genes conferred a similar sensitivity to drought when overexpressed in *Arabidopsis;* in contrast, the *pub22 pub23* double knockout mutants displayed increased drought tolerance [24]. PUB22 and PUB23 also represent good examples of how complex PUB cellular functions can be as these proteins have also been implicated as negative regulators of plant defence responses. The *pub22 pub23 pub24* triple knockout mutant was found to display increased resistance to both bacterial and oomycete pathogens, and this response was accompanied by increased oxidative bursts and cell death [30]. Similarly, the potato PUB17 protein was implicated in both biotic and abiotic stresses where the RNAi-supression of PUB17 resulted in potato plants with increased susceptibility to a *Phytophthora infestans* pathogen and increased sensitivity to salt stress [25].

A further connection with PUB44 and ABA was observed with the plasma membrane localization of PUB44 in tobacco BY-2 cells with ABA treatment. PUB44 was previously identified in a plasma membrane proteomics dataset SUBA II database; [38], [39]. Recently, Drechsel et al. [43] have reported that PUB44 is localized to the plasma membrane, and that the C-terminal ARM repeats are required for this localization. As well, PUB44 was reported in the PhosPhAt database to have a phosphorylated threonine T776 in the C-terminal region [44] raising the possibility that PUB44 is interacting with a kinase at the plasma membrane. Several PUB-ARM proteins have already been found to interact with kinase domains from receptor kinases through the ARM domains [12], [13], [14], [15]. We observed that MJ, 2,4-D and ACC treatment also resulted in the plasma membrane localization of PUB44, though at a lower frequency. Similarly, the co-expression of the MLPK or ARK2 kinases with PUB44 re-localized PUB44 to the plasma membrane. This was rather unexpected as our previous observations with PUB proteins have typically yielded more specific effects. For example, PUB9 was found to re-localize to the plasma membrane when treated with ABA and ACC at a lower frequency, but not with other hormone treatments. PUB9 also re-localized to the plasma membrane when co-expressed with ARK1 kinase domain, but not when co-expressed with either MLPK or ARK2 [14]. Interestingly, we observed PUB44 localizing to the nucleus in some of the MJ or 2,4-D treated cells, as well as the MLPK and ARK2 co-expressing cells, indicating that PUB44 may also shuttle to the nucleus under certain conditions. Other PUB-ARM proteins, such as PHOR1, ARC1, PUB9, and PUB13, have been found to be present in the nucleus under certain conditions [14], [18], [21]. The results seen here may indicate that PUB44 participates in a number of different cellular responses where it localizes to the plasma membrane or possibly to the nucleus for its functions. COP1 is a well-known example of an E3 ligase whose activity is regulated by changes in its subcellular distribution: COP1 moves between the cytosol and the nucleus, and is required in the nucleus for its function [45]. One common function for PUB44 may be to regulate its activity at the plasma membrane in its negative regulation of cell death, since ABA, Jasmonic acid, and ethylene are able to promote leaf senescence. However, auxin has an opposite effect and delays leaf senescence [46], [47].

In conclusion, cell death lesions were observed through *PUB44/pub44* and *pub44/pub44* seedlings, and these haploinsufficiency effects suggest that PUB44 levels are tightly-regulated. Seeds from *PUB44/pub44* plants were also able to germinate under what would normally be inhibitory conditions indicating that PUB44 is required in *Arabidopsis* seeds to detect inhibitory germination cues. Finally, when expressed in BY-2 cells under different conditions, PUB44 was found to localize to the plasma membrane or the nucleus indicating that PUB44 can shuttle to different compartments in the cell. Future research on identifying PUB44 interactors and where they are located in the cell will help to better understand how PUB44 functions within the cell and during germination and seedling growth.

## Materials and Methods

### PCR analysis of PUB44 and PUB43 T-DNA insertion lines

The *Arabidopsis* Gene identifier number for PUB44 is At1g20780 and for PUB43 is At1g76390. SALK T-DNA insertion mutants [48] were obtained from the *Arabidopsis* Biological Resource Center Ohio State, U.S.A, and the SAIL Syngenta *Arabidopsis* Insertion Library mutant was acquired through Syngenta [49]. Insertion mutant information was obtained from the SIGnAL website at http://signal.salk.edu, and from the TMRI website (no longer available). For primers used in the PCR reactions with genomic DNA or cDNA, all primer sequences are listed in Table S1. The *pub44-2* allele SALK_076799 and *pub43-1* allele SALK_112870 were identified by genomic PCR using a gene specific primer and the LBa1 T-DNA left border primer while the *pub44-1* allele Sail_716_H08 was identified by genomic PCR using a gene specific primer and the LBa3 T-DNA left border primer. For RT-PCR analysis of the T-DNA insertion lines, RNA was extracted using Trizol reagent Invitrogen; DNase treated, and then reverse-transcribed using the SuperScriptII First-Strand cDNA Synthesis kit Invitrogen. The cDNA was then used as a template for PCR reactions using primers (Table S1) shown in Figure 1.

### Plant Materials and Growth Conditions

For plants grown on soil, seeds were first sterilized and stratified at 4°C for 3 days in the dark, then sown on soil supplemented with 20-20-20 fertilizer. For routine plant growth, plants were grown under long days 16 hours light and 8 hours dark, 22°C and light levels of 125 µmol m^−2^ s^−1^. For pathogen infection experiments, plants were grown under short days 8 hours light, 16 hours dark with light levels at 150 µmol m^−2^ s^−1^. Under either of these growth conditions, the *pub44-1* and *pub44-2* mutants do not survive due to a seedling lethal phenotype. The very high-light rescue conditions were not known at the time [22], and so all experiments were conducted on seeds from heterozygous *PUB44/pub44-1* and *PUB44/pub44-2* plants.

For the plate germination assays, seeds were sterilized, sown on agar plates containing ½ Murashige and Skoog salts and 0.05% MES, and stratified at 4°C for 3 days in the dark. Plates were then transferred to a 16 hour light/8 hour dark photoperiod at 22°C with light levels of 75 µmol m^−2^ s^−1^ in the Percival series 101 growth chamber. When required, the plate media was supplemented with sucrose, abscisic acid ABA, NaCl, or mannitol as described in the results. All lines were grown together at the same time prior to harvesting the seeds used for these germination experiments.

### Pathogen infections

Bacterial infection was performed by injecting *Pseudomonas syringae* pv. tomato DC3000 at a concentration of 5×10^5^ CFU ml-1 in 10 mM MgCl_2_ into the abaxial surface of *Arabidopsis* leaves from 4 week old plants. At 0 and 3 dpi, leaf discs were collected, homogenized in 10 mM MgCl_2_ and plated on King's B medium agar plates after appropriate dilution. After incubation at 28° for two days the number of colonies was determined. Infection with *Hyaloperonospora arabidopsidis* isolate Noco2 was performed by applying a single drop of asexual inoculum suspension 1×10^5^ conidiosporangia/mL per cotyledon of 7-d-old seedlings. The seedlings were grown at 16°C and >90% RH with an 8-h photoperiod. Plants whose leaves displayed sporangiophores were scored as susceptible at 7 d after inoculation [50].

### PUB44 and PUB43 Promoter GUS analyses

For the PUB44_pro_GUS construct, the 5′ upstream region of 1377 base pairs from the predicted ATG was PCR-amplified from genomic DNA and cloned into HindIII/NcoI sites of the pCambia1391Z vector. For the PUB43_pro_GUS construct, the 5′ upstream region of 1163 base pairs from the predicted ATG was PCR-amplified from genomic DNA and cloned into the EcoR1 site of the pCambia1391Z vector. Transgenic *Arabidopsis* were created via the floral dip method [51] using Agrobacterium GV3101. T1 seeds were screened on agar plates with ½ MS and 50 mg/ml hygromycin.

For the GUS analysis, T3 seedlings and plants were used. Seedlings or plant material were harvested and submerged in a solution of 100 mM NaPO4 pH 7, 100 mM EDTA pH 8, 0.5 mM Potassium Ferricyanide, 0.5 mM Potassium Ferrocyanide, 0.1% Triton, 1.9 mM X-GLUC 5-bromo-4-chloro-3-indolyl-beta-D-glucuronic acid and 20% methanol. The tissues were then vacuum-infiltrated for 45 minutes at room temperature in the dark, and then incubated at 37°C for colour development. Following this, the buffer was removed and replaced with 100% ethanol for tissue clearing. Once tissues were free of chlorophyll, 8∶2∶1 w:v:v chloral hydrate:water:glycerol was added for further clearing and for mounting. Pictures were taken on the Leica MZ16 scope and IM50 software. For seedling treatments, seedlings were harvested from ½ MS plates either at 7 days post germination and treated with 10 µM or 50 µM abscisic acid in ½ MS liquid media for 2 hours, shaking at room temperature prior to staining.

### Transient expression and subcellular localization studies in BY-2 cells

Tobacco BY-2 cells were transformed via biolistic bombardment as previously described [14], [18].The entire coding region for PUB44 was cloned into the XbaI site of pRTL2 to produce a GFP-tagged protein. The kinase domain from the ARK2 receptor kinase was cloned as a GST-fusion while MLPK has a C-terminal MYC tag as previously described Samuel et al. 2008. These constructs were then bombarded into tobacco BY-2 cells and transiently expressed for 16–18 hours. Cells were fixed with 4% paraformaldehyde and visualized either directly through fluorescent microscopy for GFP or first incubated with either rabbit anti-GST or mouse anti-MYC antibodies as previously described Stone et al. 2003. Cells were visualized with the Axioskop 2 MOT epifluorescence microscope Carl Zeiss. For phytohormones treatments, BY-2 cells transiently expressing GFP:PUB44 were first treated with abscisic acid (ABA), gibberellic acid (GA_3_), methyl jasmonate (MJ), 2,4-dichlorophenoxyacetic acid (2,4-D), or 1-aminocyclopropane-1-carboxylic acid (ACC) at concentrations of 10 µM in 0.1 mN NaOH for 2 hours followed by fixation and epifluorescence microscopy.

## Supporting Information

Table S1 Primers used in study.
(DOCX)
