# Supplementary material for: Altered Germination and Subcellular Localization Patterns for PUB44/SAUL1 in Response to Stress and Phytohormone Treatments
Source: PLoS One. 2011 Jun 27;6(6):e21321. doi: 10.1371/journal.pone.0021321 (PMC3124495; doi:10.1371/journal.pone.0021321)
Supplement: Table S1 — Primers used in study. (DOCX) [file pone.0021321.s001.docx]

**Table S1 – Primers used in study.**

| Name | Sequence |
| --- | --- |
| LBa1 | TTTTTCGCCCTTTGACGTTGGA |
| LBa3 | CATCTGAATTTCATAACCAATCT |
|  |  |
| PUB44 44-1 | GGTAGACTCCAGCCTCTTCTAGG |
| PUB44 44-2 | TAGCTCTCATTTTGAGCGAGG |
| PUB44 P1f | TTCTTATCACATGAGCCATCAAA |
| PUB44 P1r | AATCATCATTCTCTCGGACCT |
| PUB44 P2f | GCCAAGGGGATATCAAGGGA |
| PUB44 P2r | GCGTTCTCAGCGATCTGTCT |
| PUB44 P3f | ATGGTTGGAAGCTCGGATGG |
| PUB44 P3r | GCTCTCATCATCTCCTTCAAC |
| PUB44 P4f | GAGAATGGTCGAACATTCGAG |
| PUB44 P4r | GAAAGGAAGCCATACACACTT |
| PUB44 promoterFOR | GCGGCGAAGCTTCGAAATTTAGCTCCAACACACACGCGA |
| PUB44 promoterREV | GCGGCGCCATGGCTTAATCCTTCAAACTCAACTGCAAATG |
|  |  |
| PUB43 P1f | CGTGTGTGCAATCCTCAGC |
| PUB43 P1r | TTGGAGAAGGTTCTCCACTAT |
| PUB43 P2f | TCCAAACTCGGTTATTAATGTTG |
| PUB43 P2r | ATCTCACTCCTTGTACAACATC |
| PUB43 P3f | ATGGCTGGAAGTGGAAGTTG |
| PUB43 P3r | CTTCAAGAAGTTTAGCAAGAAGA |
| PUB43 promoterFOR | GCGGAATTCTCTCTCTCTTCTCTTGTCTCTCTC |
| PUB43 promoterREV | GCGGAATTCCTGATGAATTGATTCTTCTACACCAT |
